# Quantification of 11 metabolites in rat urine after exposure to organophosphates

**DOI:** 10.1186/s42826-024-00209-3

**Published:** 2024-06-06

**Authors:** Michael A. Leninsky, Vladislav E. Sobolev, Margarita O. Sokolova, Natalya G. Voitenko, Nikita V. Skvortsov

**Affiliations:** 1 Sechenov Institute of Evolutionary Physiology and Biochemistry, Russian Academy of Sciences, Thorez 44, St. Petersburg, 194223 Russia; 2 Federal State Budgetary Scientific Institution “Institute of Experimental Medicine”, 12, Acad. Pavlov Street, St. Petersburg, 197022 Russia

**Keywords:** Organophosphates, Poisoning, Rat, Metabolites, Urine, Paraoxon-ethyl, 2-(o-cresyl)-4H-1,3,2-benzodioxaphosphorin-2-oxide, Chromatography-mass spectrometry

## Abstract

**Background:**

The aim of the study was to develop a technique for quantitative determination of rat urine metabolites by HPLC–MS/MS, which can be used to search for biomarkers of acute intoxication with organophosphates (OPs).

**Results:**

The content of metabolites in the urine of rats exposed to a single dose of paraoxon (POX1x); interval, twice daily administration of paraoxon (POX2x); exposure to 2-(o-cresyl)-4H-1, 3, 2-benzodioxaphosphorin-2-oxide and paraoxon (CBPOX) was investigated. New data were obtained on the content in the urine of intact rats as well as rats in 3 models of OP poisoning: 3-methylhistidine, threonine, creatine, creatinine, lactic acid, acetylcarnitine, inosine, hypoxanthine, adenine, 3-hydroxymethyl-butyrate and 2-hydroxymethyl-butyrate.

**Conclusions:**

The proposed assay procedure is a simple and reliable tool for urine metabolomic studies. Within 1–3 days after OP exposure in all three models of acute intoxication, the concentration of metabolites in rat urine, with the exception of adenine, changes similarly and symmetrically, regardless of the method of poisoning modeling, in all three models of acute intoxication. Further studies are needed to determine the specificity and reliability of using urinary metabolite concentration changes as potential biomarkers of acute organophosphate intoxication.

**Graphical Abstract:**

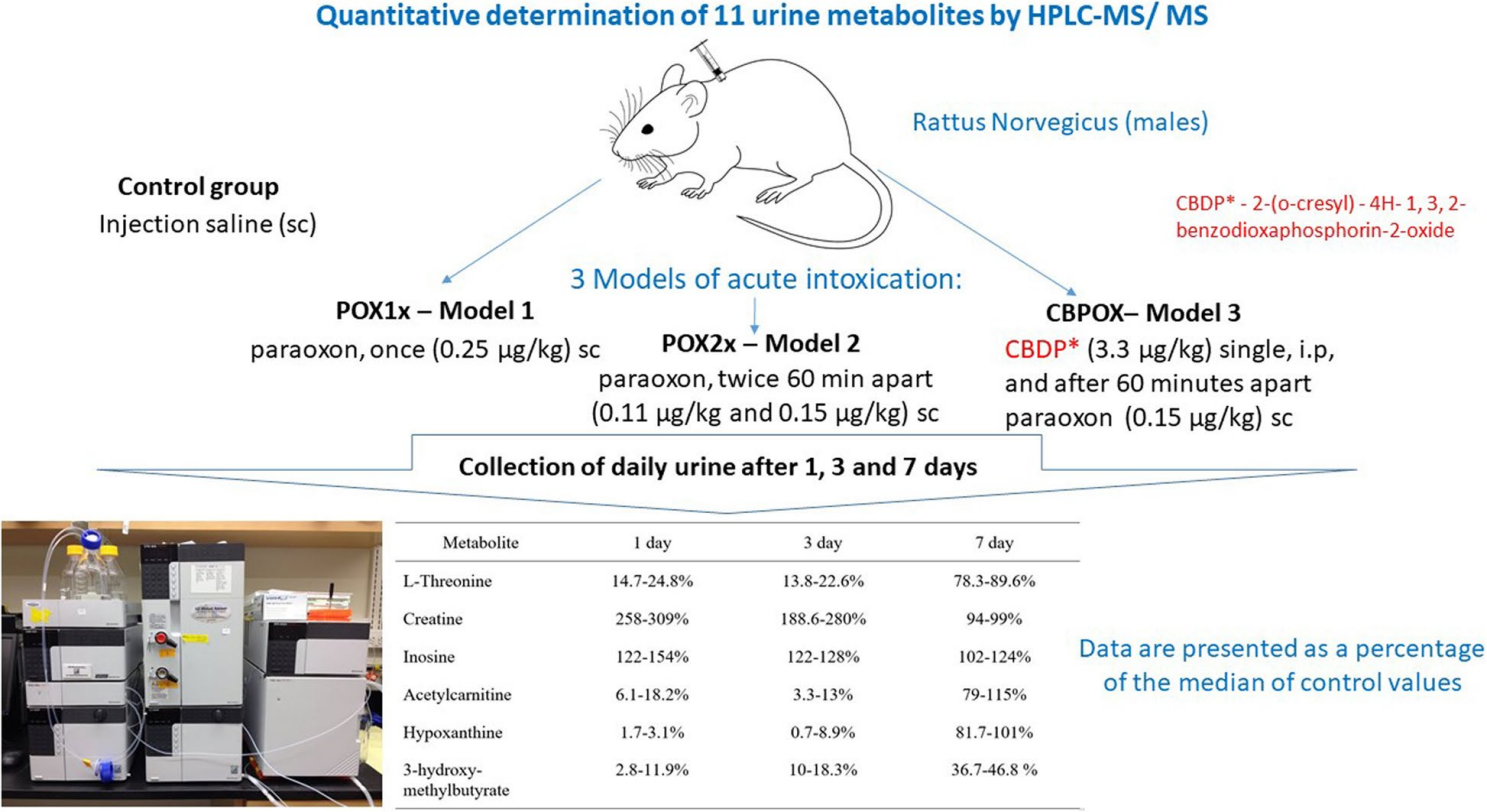

**Supplementary Information:**

The online version contains supplementary material available at 10.1186/s42826-024-00209-3.

## Background

The clinical manifestations of acute human exposure to OP can be accurately reproduced in rodents and non-human primates. Guinea pigs and nonhuman primates, like humans, have low levels of circulating carboxylesterases and are therefore the most appropriate animal models for studying OPs intoxication [1]. Laboratory rats are also used in experiments with OPs [1]. However, this species has physiological peculiarities, in particular the presence of high plasma carboxylesterase (CE) activity. In this regard, suppression of blood plasma CE activity in rats may improve the adequacy of experimental models in studying the mechanism of action of OPs.

In 2019, Goncharov N.V. et al. developed 2 models of acute OP poisoning in rats, with preliminary inhibition of carboxylesterases by administration of 2-(o-cresyl)-4H- 1, 3, 2-benzodioxaphosphorin-2-oxide (CBDP) or fractional administration of total dose of studied OPs [2]. The above models allow effective evaluation in rats of various aspects of OPs toxicity comparable to human poisoning, including effects on blood biochemistry, nervous system, cardiovascular system and renal function [1, 3–5].

In modern diagnostics, the importance of non-invasive procedures for assessing functional status, including the analysis of metabolite content in body fluids, is increasing. It is therefore of interest to evaluate the prospects of using urinary metabolites as biomarkers of OPs exposure using three models of poisoning. In addition, for most of the 11 metabolites we selected, there is no information on changes in their concentrations in the urine of rats after acute exposure to organophosphate.

The objective of this study was to develop a comprehensive methodology for the analysis of metabolites in rat urine in three models of acute OP poisoning. To achieve this goal, we employed high-performance liquid chromatography with tandem mass spectrometric detection (HPLC–MS/MS).

The metabolites in the present study were selected after analyzing the existing techniques for their determination in urine. The content of 3-methylhistidine in human urine is used to assess muscle protein metabolism and degradation [6]. In this regard, increased urinary excretion of 3-methylhistidine is observed in inflammatory processes. Threonine is an amino acid involved in the process of protein synthesis and the formation of other amino acids, regulates the liver, central nervous, cardiovascular and immune systems, and promotes tissue repair. Creatine is an energy substrate required for adenosine triphosphate (ATP) regeneration [7]. Creatinine, being the final product of creatine breakdown, reflects impaired renal function. Lactic acid is a product of anaerobic breakdown of glucose in muscle tissues and is responsible for the utilisation of excess nitrogen in muscle. Increased levels of lactate in the urine indicate hyperuricaemia. Acetylcarnitine promotes the transport of essential fatty acids across cell membranes and thus may play a role in normalising intracellular lipid metabolism and regulating peripheral nerve function and regeneration [8]. Inosine stimulates the synthesis of adenine nucleotides, increases the activity of some enzymes of the Krebs cycle. Inosine and adenine are considered as potential diagnostic markers of chronic atrophic gastritis [9]. Hypoxanthine is one of the breakdown products of ATP and can be considered as an indicator of reduced oxygen content in certain organs and tissues [10], as well as a marker of radioactive exposure [11]. In chronic renal failure, reabsorption of adenine may be impaired, leading to its excretion in the urine. Measurement of urinary adenine levels can be used as an indicator of impaired purine metabolism. Thus, the choice of adenine as a metabolite in rat urine is justified due to the potential effect of organophosphate compounds on renal structure and function [5]. 3-Hydroxymethylbutyrate is a metabolic product of leucine, which is responsible for protein synthesis and is obtained from food. 2-Hydroxymethylbutyrate found in the urine of patients with phenylketonuria [12], lactoacidosis and ketoacidosis [13]. 2-Hydroxymethylbutyrate is formed mainly by ketogenesis and metabolism of valine, leucine and isoleucine [14]. The list of urine metabolites is presented in Table 1.

**Table 1:** List of metabolites selected for analysis of their content in rat urine

Metabolites	Gross formula	Molecular weight, Da	Structural formula
3-Methylhistidine	C_7_H_11_N_3_O_2_	169.181	
Threonine	C_4_H_9_NO_3_	119.119	
Creatine	C_4_H_9_N_3_O_2_	131.133	
Creatinine	C_4_H_7_N_3_O	113.118	
Lactic acid	C_3_H_6_O_3_	90.078	
Acetylcarnitine	C_9_H_17_NO_4_	203.236	
Inosine	C_10_H_12_N_4_O_5_	268.226	
Hypoxanthine	C_5_H_5_N_5_	136.112	
Adenine	C_10_H_13_N_5_O_4_	135.127	
3-Hydroxymethylbutyrate	C_5_H_10_O_3_	118.131	

## Methods

### Animals

The study was conducted on male rats (Rattus norvegicus) of the outbred Wistar line, weighing 200–240 g. The animals were housed in cages and maintained in rooms with controlled temperature and humidity, with a 12-h light–dark cycle and free access to food and water. All animal housing, experimental procedures, and manipulations were conducted in strict accordance with the requirements of the national bioethics guidelines and in alignment with the recommendations of Directive 2010/63/EC of the European Parliament and of the Council of the European Union of 22 September 2010 on the protection of animals used for scientific purposes and the European Convention for the Protection of Vertebrate Animals Used for Experiments or Other Scientific Purposes. The Bioethics Committee of the I.M. Sechenov Institute of Evolutionary Physiology and Biochemistry of the Russian Academy of Sciences granted permission to conduct experiments with animals. Ethical approval number 13-k-a, dated 15 February 2018.

### Materials

Acetonitrile for high-performance liquid chromatography was purchased from Panreac (Spain), HPLC grade methanol (J.T. Baker, Netherlands), and ammonium formate (Acros organics, Belgium). Paraoxon, formic acid, creatinine, creatine, acetylcarnitine hydrochloride, hypoxanthine, threonine, inosine, lactic acid, 3-Methylhistidine, 2-hydroxymethylbutyrate, 3-hydroxymethylbutyrate and paraoxon-ethyl were purchased from "Sigma-Aldrich" (USA). Deuterated (D-3) 2-(2-carboxyethyl)-1,1,1-trimethylhydrazinium (purity 99.9%), 2-(o-cresyl)-4H-1,3,2-benzodioxaphosphorin-2-oxide (purity 99.5%) were synthesised by the laboratory staff, the purity of the obtained substances was confirmed by NMR spectroscopy and HPLC–MS/MS.

### Experiment

Experiments on modeling of intoxication of rats were carried out in the Research Institute of Hygiene, Occupational Pathology and Human Hygiene and Ecology of the Federal Medical and Biological Agency of Russia in 2016–2018 within the framework of the project of Russian Science Foundation № 16–15-00199 "Development of effective means of prevention, therapy and prevention of delayed consequences of poisoning by organophosphorus compounds". In this study rats were divided into 4 groups of 6 animals each. The computer program Research randomizer (https://randomizer.org) was used for randomisation when forming the groups. The method of toxicant administration was a single subcutaneously injection. In the present study, three animal poisoning models were used: 1. Single injection of paraoxon (POX) at LD50 dose (POX1x); 2. Fractional injection of POX at doses of 110 µg/kg + 150 µg/kg, 60 min apart; 3. Single injection of CBDP inhibitor (3.3 µg/kg) and POX (0.15 µg/kg) at 60 min apart (Table 2). Control group rats was treated with physiological solution subcutaneously. The study duration was 7 days. Daily urine samples were collected in Techniplast® Metabolic cages for mice and rats (Braintree Scientific, USA) to which the animals were pre-adapted to prevent stress.

**Table 2:** Animal groups (models of acute OP exposure) and doses of paraoxon and other compounds

Group	Substance administered (dose)
Control	Injection saline
POX1x – Model 1	Paraoxon, once (0.25 µg /kg)
POX2x – Model 2	Paraoxon, twice 60 min apart (0.11 µg /kg and 0.15 µg /kg)
CBPOX– Model 3	Single administration of CBDP (3.3 µg /kg) and paraoxon (0.15 µg /kg) 60 min apart

Daily urine samples were collected before OP administration (background) and on days 1, 3 and 7 after poisoning. Samples were frozen and stored at (- 18 ºC) until analysis.

### HPLC–MS/MS analysis

To evaluate the effects of POX and CBDP in rats, a series of biochemical parameters were selected for determination in urine.

Samples were analysed using a Shimadzu LC-20 liquid chromatograph equipped with a Shimadzu LCMS-8050 electrospray ionisation (ESI) mass spectrometric detector. Data processing was performed using the Labsolution "Quant browser".

Chromatographic separation was performed using a Zorbax SB-C8 column (Agilent), length 150 mm, column diameter 4.6 mm, sorbent particle diameter 1.8 μm. Mobile phase: component A—solution of 0.1% formic acid and 10 mM ammonium formate in deionised water; component B—solution of 0.1% formic acid and 10 mM ammonium formate in methanol. Eluent flow rate: 0.4 cm3/min. Column thermostat temperature: 40°C. Sample compartment thermostat temperature: 5°C. Sample injection volume: 5 µl. The elution programme was as follows: 0.0—1.0 min 5% B; 1.0—7.0 min 5—90% B; 7.0—10.0 min 90% B; 10.1—15.0 min 5% B.

Mass spectrometric detection was performed in multiple reaction monitoring (MRM) mode. Flow rate: desiccant gas—10 dm^3^/min; auxiliary gas—10 dm^3^/min. Atomiser pressure, 3 dm^3^/min; desiccant gas temperature, 300 ºC; auxiliary gas temperature, 350°C; capillary voltage, 3500 V; fragmenter voltage, 120 V.

**Fig. 1:**
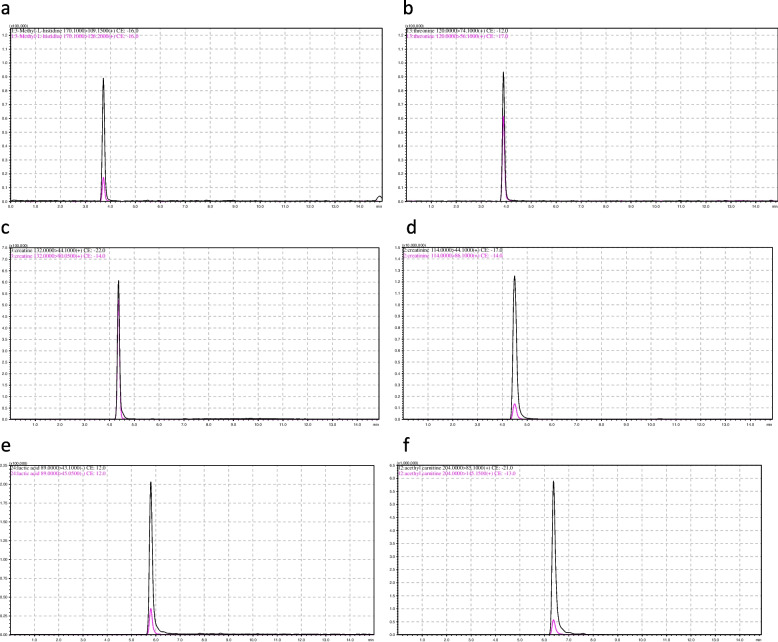
Mass chromatograms of metabolites in rat urine: **a** 3-methylhistidine; **b** L-threonine; **c** Creatine; **d** Creatinine; **e** Lactic acid; **f** Acetylcarnitine. An additional file shows this in more detail (see Additional file 1)

**Fig. 2:**
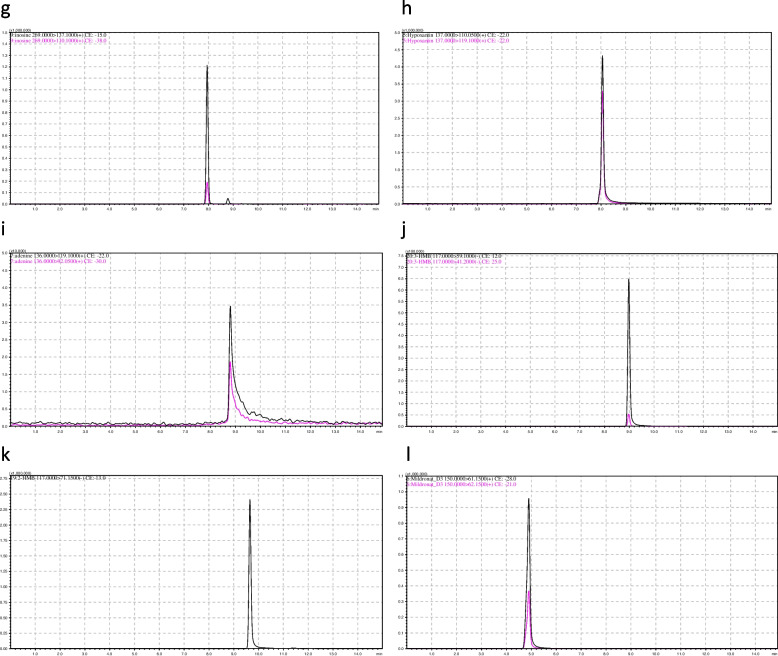
Mass chromatograms of metabolites in rat urine: **g **Inosine; **h **Hypoxanthine; **i **adenine; **j **3-hydroxybutyrate; **k **2-hydroxybutyrate; **l **Deuterated (D-3) 2-(2-carboxyethyl)-1,1,1,1-trimethylhydrazinium. An additional file shows this in more detail (see Additional file 2)

The retention times and MRM transitions (precursor-ion > product-ion) are presented in Table 3. The chromatograms of the metabolites are shown in Figs. 1 and 2.

**Table 3:** Retention times and MRM transitions of the investigated metabolites

Indicator (ionisation)	Rt, min	MRM transition (precursor-ion > quantitative/qualitative product-ions)
3-methylhistidine( +)	3.8	170.1 > 109.15 / 126.2
Threonine( +)	4.0	120.0 > 74.1 / 56.1
Creatine( +)	4.5	132.0 > 44.1 / 90.05
Creatinine( +)	4.6	114.0 > 44.1 / 86.1
Lactic acid(-)	6.1	89.0 > 43.1 / 45.05
Acetylcarnitine( +)	6.5	204.0 > 85.1 / 145.15
Inosine( +)	8.1	269.0 > 137.1 / 110.1
Hypoxanthine( +)	8.3	137.0 > 110.1 / 119.1
Adenine( +)	8.9	136.0 > 119.1 / 92.05
3-Hydroxy methylbutyrate (-)	9.3	117.0 > 59.1 / 41.2
2-Hydroxy methylbutyrate (-)	10.0	117.0 > 71.15
D3-2-(2-carboxyethyl)-1,1,1,1-trimethyl hydrazinium ( +)	4.9	150.0 > 61.15 / 62.15

To assess the suitability of the methodology, the inter-run accuracy and precision of the results obtained were evaluated. Controls were performed within 4 cycles at the concentration levels of each analyte: lower limit of quantification, mid-range, and concentration near the upper limit of the quantification range.

### Sample preparation for analysis

Due to the fact that the detectable levels of the metabolites in rat urine were quite high, sample preparation was reduced to the dilute-n-shoot technique.

Sample preparation was carried out as follows. A 2.0 cm^3^ Eppendorf tube was filled with 0.3 cm^3^ of urine sample and 0.9 cm^3^ of acetonitrile containing D3-2-(2-carboxyethyl)-1,1,1,1-trimethylhydrazinium used as an internal standard (IS). The resulting sample was diluted tenfold and 1000-fold. Similar preparation was carried out in parallel for blank urine sample.

### Statistics

Data processing was performed in GraphPad Prism 8.4.3. The data were checked for normality of distribution (Kolmogorov–Smirnov test). Due to the small size of the groups (less than 10 animals), nonparametric statistics was used. Data are presented as medians and 5–95 percentiles (descriptive statistics). One-factor analysis of variance (Kruskal–Wallis test) and Dunn's multiple comparisons test (Dunn's multiple comparisons test) for multiple comparisons (comparison of each background with the "control") were used to compare background values of metabolites (comparison of each background with the "control"). Two-factor analysis of variance (2way ANOVA) and Dunnett's multiple comparisons test (Dunnett's multiple comparisons test) were used to evaluate the effect of time elapsed after exposure and intoxication pattern. Results were considered statistically significant at *p* < 0.05.

## Results

### HPLC–MS/MS analysis

The values of the achieved metrological performance of the developed technique are presented in Table 4. The standards of accuracy (85–115%) and precision (< 15%) were met, indicating the stability of the chosen analysis technique.

**Table 4:** Analytical characteristics of the methodology for the determination of urine metabolites

Urine metabolites	Measuring range, µMol/L	Detection accuracy (between cycles)	Detection precision (between cycles)
3-methylhistidine	0.3—5.9	92.6—110.2%	7.4—10.9%
L-threonine	4.2—83.9	88.1—109.9%	5.4—11.7%
Creatine	0.4—7.6	90.7—108.1%	6.1—14.5%
Creatinine	4.4—88.4 mMol/L	86.7—113.1%	6.4—11.6%
Lactic acid	55.5—1110.1	87.7—108.9%	7.0—11.1%
Acetylcarnitine	24.6 – 492.0	89.5—105.2%	5.2—13.2%
Inosine	1.9—37.3	89.2—108.7%	6.9—11.7%
Hypoxanthine	18.4—367.3	88.1—108.7%	5.1—12.5%
Adenine	18.5 – 370.0	86.3—110.3%	6.1—11.0%
3-Hydroxymethylbutyrate	21.2—423.3	87.3—111.4%	7.7—10.3%
2-Hydroxymethylbutyrate	21.2—423.3	89.0—110.7%	7.1—14.4%

Comparison of the results of metabolites content determination in the urine of rats. Prior to the study, daily urine was collected from rats of all groups and analysed according to the described methodology to establish background levels of biochemical parameters in the absence of exposure to OPs and other stress factors (Table 5).

**Table 5:** Metabolite content in rat urine before exposure to OP (*n* = 6). Data are presented as median and 5–95 percentile interval

Metabolite	Concentration, μMol/L			
	Control	POX1x	POX2x	CBPOX
3-Methyl-histidine	1.10 (0.92; 1.21)	1.10 (1.02; 1.13)	1.27 (0.92; 1.34)	1.03 (0.74; 1.51)
L-Threonine	33.3 (29.8; 35.3)	33.5 (31.6; 38.5)	32.8 (29.8; 32.9)	32.2 (31.9; 32.7)
Creatine	1.21 (1.19; 1.30)	1.21 (1.21; 1.32)	1.29 (1.15; 1.31)	1.20 (1.19; 1.23)
Creatinine (mMol/L)	4.16 (4.10; 4.17)	4.21 (3.39; 4.95)	4.26 (3.75; 4.32)	4.51 (3.75; 4.64)
Lactic acid	656 (649; 657)	649 (591; 670)	636 (618; 666)	650 (647; 670)
Acetyl carnitine	330 (330; 360)	349 (344; 380)	404 (191; 563)	258 (237; 445)
Inosine	5.40 (5.00; 6.50)	5.25 (4.50; 7.30)	4.90 (4.80; 5.90)	5.80 (4.70; 5.90)
Hypoxanthine	54.0 (51.8; 58.4)	54.0 (36.0; 69.0)	58.3 (53.5; 62.2)	54.7 (53.8; 61.3)
Adenine	36.7 (35.7; 37.5)	33.3 (13.0; 88.0)	37.3 (37.1; 44.7)	35.6 (35.2; 37.4)
3-hydroxy-methylbutyrate	112 (109; 113)	121 (107; 132)	106 (101; 115)	108 (107; 116)
2-hydroxy-methylbutyrate	67.0 (60.8; 72.0)	64.1 (60.8; 75.8)	65.7 (62.6; 67.3)	65.0 (61.8; 67.9)

One-factor analysis of variance (one-way ANOVA) revealed no statistically significant differences between the background values of the groups, indicating correct randomisation of laboratory animals. To gain insight into the physiological fluctuations of metabolite concentrations in rat urine, all background values and values of control animals at different times of the experiment were combined into a large control group (*n* = 42). The spread of values from minimum to maximum was taken as a normal (or physiological) interval (indicated by the dotted line in the figures with the dynamics of changes after the administration of toxicants).

In one day after exposure to OPs, a rather significant decrease in the concentration of acetylcarnitine, threonine, isomers of 3-hydroxymethylbutyrate and hypoxanthine in urine was observed (almost 50-fold decrease). Also, one day after poisoning, an increase in urine creatinine (by 30%), creatine (2.5-fold), lactic acid (threefold) and inosine was noted. The increase in inosine content was uneven depending on the group—about 10% for POX1x, 50% for POX2x, 40% for CBPOX.

On the third day after poisoning, a decreased concentration of 3-methylhistidine (at 50%); threonine, acetyl-carnitine; hypoxanthine and 3-hydroxymethylbutyrate was still observed in the urine of rats. The concentration of creatine and creatinine in urine remained elevated twofold or more in all groups of rats exposed to OPs, regardless of the poisoning model used. On the seventh day after exposure to OP, the content of almost all metabolites in the urine of rats stabilised and did not differ from background and control values, except for threonine and 3-hydroxymethylbutyrate, the concentrations of which remained significantly reduced.

**Fig. 3:**
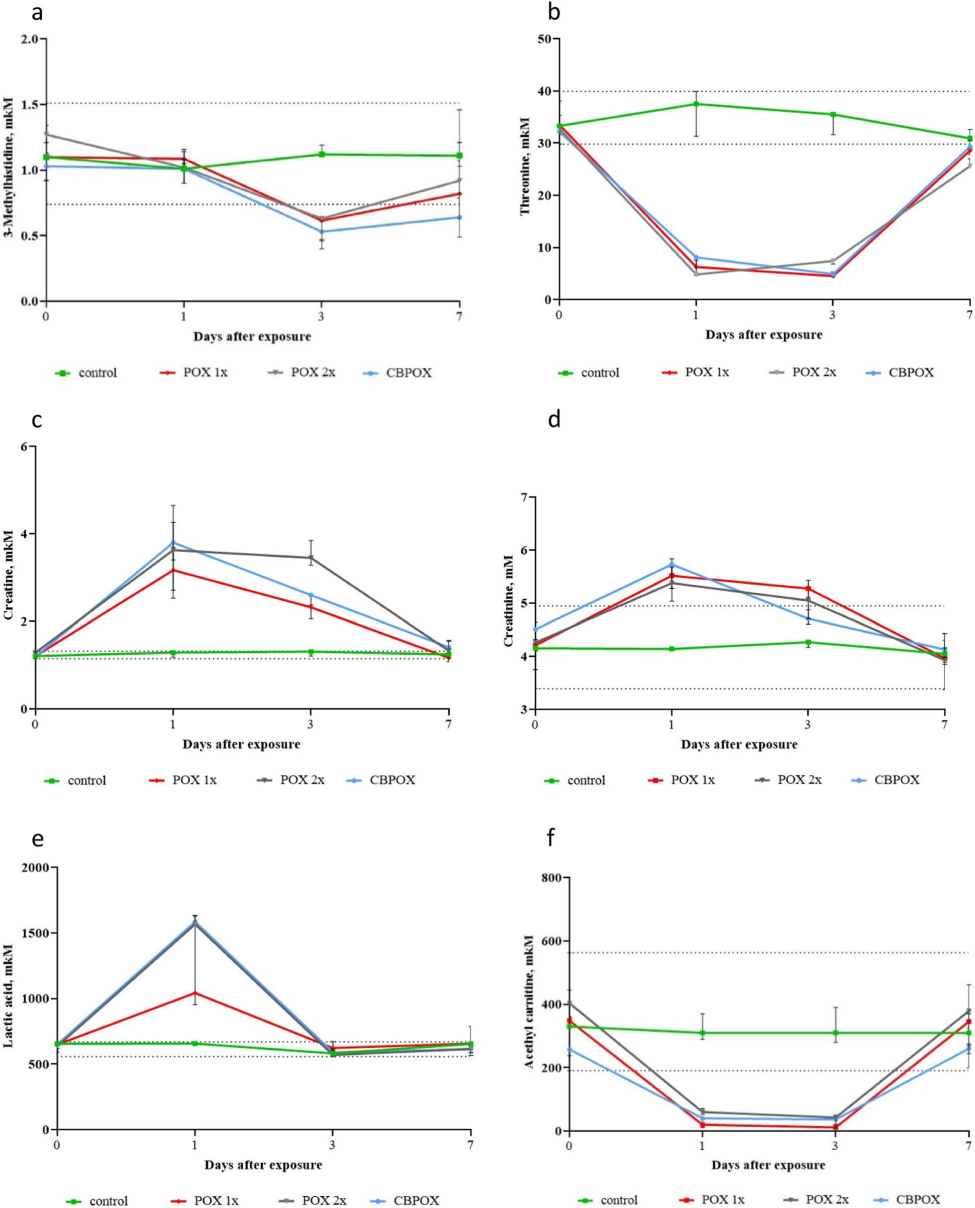
Dynamics of metabolite content changes in rat urine: **a** 3-methylhistidine; **b** Threonine; **c** Creatine; **d** Creatinine; **e** Lactic acid; **f** Acetyl-carnitine. The vertical bars at each point on the graph denote the interquartile range. An additional file shows this in more detail (see Additional file 3)

**Fig. 4:**
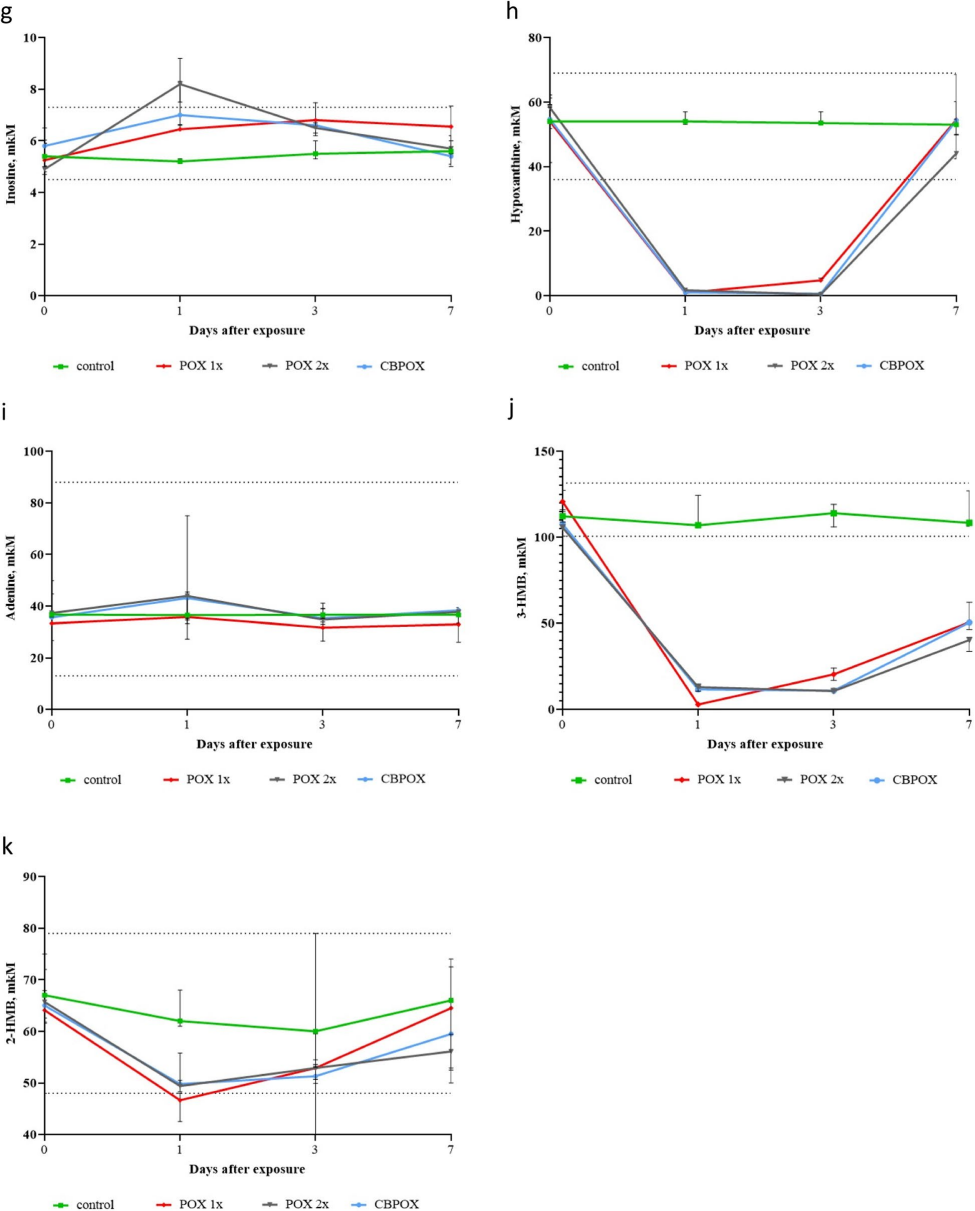
Dynamics of changes in the content of metabolites in rat urine: **g **Inosine; **h **Hypoxanthine; **i **Adenine; **j **3-hydroxymethylbutyrate (3-HMB); **k **2-hydroxymethylbutyrate (2-HMB). The vertical bars at each point on the graph denote the interquartile range. An additional file shows this in more detail (see Additional file 4)

Figures 3 and 4 show the dynamics of changes in the concentration of the analysed metabolites in urine at different times after OPs exposure. In these figures, each point corresponds to the median, and the dotted line indicates the physiological interval of change in metabolite concentration according to the data of the pooled control (*n* = 42).

The data presented in Table 6 also allow us to draw three main intermediate conclusions:

**Table 6:** Two-factor analysis—effect of 3 models of OP poisoning and time after administration on the concentration of metabolites in rat urine

	Models of poisoning (POX1x; POX2x; CBPOX)	Time after exposure	Interaction of factors
3-Methyl-histidine	*P* = 0,0433	*P* < 0,0001	*P* < 0,0001
L-Threonine	*P* < 0,0001	*P* < 0,0001	*P* < 0,0001
Creatine	*P* < 0,0001	*P* < 0,0001	*P* < 0,0001
Creatinine (mMol/L)	*P* = 0,001	*P* < 0,0001	*P* < 0,0001
Lactic acid	*P* < 0,0001	*P* < 0,0001	*P* < 0,0001
Acetyl carnitine	*P* < 0,0001	*P* < 0,0001	*P* < 0,0001
Inosine	*P* = 0,001	*P* < 0,0001	*P* < 0,0001
Hypoxanthine	*P* < 0,0001	*P* < 0,0001	*P* < 0,0001
Adenine	*P* = 0,421	*P* = 0,265	*P* = 0,870
3-hydroxy-methylbutyrate	*P* < 0,0001	*P* < 0,0001	*P* < 0,0001
2-hydroxy-methylbutyrate	*P* = 0,052	*P* < 0,0001	*P* = 0,001

Adenine should be excluded from further consideration for the search of biomarkers of OPs intoxication. The used models of exposure to OP and time after administration do not affect the concentration of this metabolite in rat urine.

For all other metabolites mutual influence of factors (time and model of OP administration) is observed, which was the expected result.

The effect of observation time after OP administration on the concentration of metabolites is more pronounced than the intoxication model (for 3-methylhistidine, creatinine, and inosine), and only time (but not the nature of the toxicant) affects the concentration of 2-hydroxy-methylbutyrate. This is also expected since different poisoning models but not different toxicants were used for the experiment.

The results obtained in our study are interesting for diagnostics because they allow us to confidently distinguish healthy animals from those exposed to OP both immediately after exposure and after some time. Table 7 shows the direction and degree of intensity of changes in the content of metabolites in urine after exposure to toxicants.

**Table 7:** Metabolites in rat urine after exposure to OP. Data are presented as a percentage of the median of control values

Metabolite	1 day	3 day	7 day
L-Threonine	14.7–24.8%	13.8–22.6%	78.3–89.6%
Creatine	258–309%	188.6–280%	94–99%
Inosine	122–154%	122–128%	102–124%
Acetylcarnitine	6.1–18.2%	3.3–13%	79–115%
Hypoxanthine	1.7–3.1%	0.7–8.9%	81.7–101%
3-hydroxy-methylbutyrate	2.8–11.9%	10–18.3%	36.7–46.8%

Of the metabolites in Table 7, creatine is of interest from the point of view of ease of diagnosis because a significant increase in the concentration of this metabolite is easier to trace, but, on the other hand, it is difficult to operate on an increase in its concentration without an understanding of the urine volume and density obtained. It is possible that the increase in creatine concentration (as well as creatinine) is associated with a decrease in water intake in the first to third day after poisoning, which may have caused a decrease in urine volume and an increase in urine density. It should also be noted that the second metabolite contained in the urine of rats in increased concentration during 1–3 days after poisoning was inosine.

The results obtained in the present study indicate that all three models of acute intoxication of rats with paraoxon lead to significant changes in the content of ten metabolites in the urine of rats early after poisoning. For further study as potential biomarkers of acute OPs intoxication it is reasonable to use six of them, which are presented in Table 7. The developed methodology for the determination of organophosphates in urine can also be used to assess the content of similar metabolites in human urine in cases of acute organophosphorus pesticide poisoning.

## Discussion

### 3-Methyl-histidine

3-Methylhistidine (3-MH) is a metabolite produced in the body by enzymatic methylation of histidine during peptide bond synthesis and methylation of actin and myosin. 3-MH is a post-translationally modified amino acid excreted with urine [15]. In this regard, 3-MH is considered as one of the suitable biomarkers that can be used to detect high protein degradation. Its excretion level is also increased in diseases associated with muscle wasting and in the elderly [16–19]. The appearance of 3-methylhistidine in urine may serve as a marker of increased muscle protein catabolism [20].

The normal concentration of 3-MH in the urine of healthy adults is in the range of 3.63–69.27 μmol/mmol creatinine, its mean concentration in urine is 15–20 μmol/mmol creatinine, and in plasma at 2.85 μM (0.0–5.9 μM) [16].

In our study, single poisoning with OPs in all 3 models resulted in a decrease in 3-MH concentration in the urine of rats at 3 days after exposure. No differences from control values were observed 1 and 7 days after poisoning. Exposure to OPs in our studies led to the development of classical clinical signs of poisoning, including muscle fascialisations etc. In this regard, the more expected effect was an increase in urinary excretion of 3-MH, but a decrease in its excretion was observed. It is not possible to give an explain for this fact without the use of additional diagnostic tools. It seems most likely that the increase of 3-MH concentration in the urine of rats after poisoning occurred earlier, within several (2–12) hours after exposure to OPs. Similar facts were noted earlier in the study of acute poisoning of rats by monocrotophos. In this case, the 3-MH content in urine significantly increased 2.5 h after poisoning and continued to increase for 10 h after poisoning. The 3-MH level decreased, but not to normal, 24 h after poisoning [21].

### Threonine

Threonine (Thr) is used for glycine synthesis during endogenous production of L-carnitine in the liver and brain [22, 23]. There are a limited number of publications on the rate of Thr metabolism in rats, and the reference range of urinary excretion has not been established. Urinary excretion of Thr in rats under dietary intake is considered to be low and nitrogen balance becomes less positive as the L-threonine content of the diet decreases from 120 to 60–30 mg per kg body weight per day. Only 29% of Thr released from whole body protein is degraded and the remainder is resynthesized into protein [24].

In our study, 1 and 3 days after exposure to OP in all models of poisoning, the concentration of Thr in the urine of rats was significantly decreased. By 7 days after poisoning, the Thr content in urine approached the "normal" limits, remaining reduced. This fact is interesting considering that many toxicants such as lead [25], nickel [26] or hydrazine [27] in acute poisoning of rats usually lead to increased urinary excretion of L-threonine, which is a particular manifestation of aminoaciduria. Unfortunately, we were unable to find publications that evaluated urinary excretion of L-threonine under the influence of other OPs. In this regard, additional studies are needed to explain the mechanism of Thr concentration decrease in acute POX poisoning. The reference range of Thr concentrations in the urine of intact rats established in our work may be useful in this regard.

### Creatine and creatinine

The main metabolic role of creatine is to combine with the phosphoryl group via the enzyme creatine kinase to form phosphocreatine, which is used to regenerate ATP. The cyclic form of creatine, creatinine, exists in equilibrium with its tautomer and with creatine [28]. Some creatine and creatine phosphate are broken down in the body to creatinine, which is subsequently excreted by the kidneys. Endogenous creatine synthesis is a two-step process. In the first step, L-arginine:glycine amidinotransferase (AGAT) catalyses the conversion of glycine and arginine to ornithine and guanidinoacetate (GAA); guanidinoacetate methyltransferase (GAMT); and in the second catalyses the S-adenosylmethionine-dependent methylation of GAA to creatine [29]. In a rat experiment, it has been shown that GAA is produced in vivo by rat and human kidneys [29]. Kidneys require phosphocreatine and creatine, and they also express a significant amount of creatine kinases, including isoenzymes of BB-CK and u-mtCK [30].

In our study, we observed a significant symmetrical increase in urinary excretion of creatine and creatinine in all models of poisoning on days 1 and 3 after exposure to OPs. By day 7, the urinary concentrations of these metabolites returned to the reference range. Similar changes in the concentration of creatine and creatinine in the urine of rats during acute OP poisoning were described earlier. Acute poisoning of rats with soman at a dose of 0.67 × LD after 24 h has been shown to result in foci of rhabdomyonecrosis in the diaphragm, with a concomitant increase in urinary creatine excretion (300% of control) and total serum creatine kinase in serum (280% of control) [31]. The instrumentation of our study does not allow a precise determination of the origin of creatinuria and creatininuria in rats. In this regard, an explanation is possible both in terms of increased levels of protein catabolism and muscle biodegradation and increased local synthesis of these metabolites by the kidneys. Further studies involving molecular diagnostic tools are needed to answer this question.

## Lactic acid

Lactic acid is an organic acid whose molecule consists of two optical isomers, L-lactic acid and D-lactic acid. The L-isomer is the most abundant in living organisms. In animals, L-lactate is constantly formed from pyruvate under the action of the enzyme lactate dehydrogenase during fermentation during normal metabolism and exercise. Its concentration does not increase until the rate of lactate production exceeds the rate of its excretion. This is regulated by a number of factors including monocarboxylate transporters, lactate concentration, LDH isoform and tissue oxidative capacity [32].

It is lactate, not glucose, that is preferentially metabolised by neurons in the brain in mice, rats and humans [33, 34]. Lactic acid acts as a general acidogen, and its high levels in blood, urine, brain and other tissues can lead to the development of metabolic acidosis, which occurs when arterial blood pH falls below 7.35. In our study, in all 3 models of OPs poisoning, a significant increase in the concentration of lactic acid in rat urine was observed after 24 h. However, after 3 and 7 days the content of this metabolite in urine returned to the limits of physiological norm, which most likely indicates the development of metabolic acidosis in animals in the early period after exposure to OPs.

### Acetyl-L-carnitine

Acetylcarnitine is the most common natural derivative and is formed by the reaction:

$$\mathrm{acetyl}-\mathrm{CoA}\;+\;\mathrm{carnitine}\;\rightleftharpoons\;\mathrm{CoA}\;+\;\mathrm{acetylcarnitine},$$

The main function of L-carnitine is to transport activated long-chain fatty acids from the cytosol to the mitochondrial matrix, where their b-oxidation takes place. After activation of fatty acids with a CoA group, the resulting acyl-CoA groups are converted to carnitine esters, when this reaction is catalysed by carnitine palmitoyl-transferase (CPT1). Carnitine esters can then cross the membrane via an additional transporter [35, 36].

Urinary excretion levels of carnitine esters and acetylcarnitine in rats were determined in an experiment with kidney perfusion for 30 min with Krebs–Henseleit bicarbonate buffer with 5 mM glucose [37]. The 30/ ~ M (- [methyl)H] carnitine reabsorption was about 96% during the first 10 min. At a concentration of 750/ ~ M, reabsorption decreased to 40%. The tubule reabsorption maximum T_max_ was about 170 nmol/min per kidney. Thus a disproportionately high excretion of carnitine or carnitine esters formed in the kidney was found in rat urine compared with the same derivatives by ultrafiltration [37].

Unfortunately, we were unable to find published studies evaluating changes in acetylcarnitine excretion with urine in animals and humans under the influence of OPs. It is known that carnitine homeostasis in the body depends on glomerular filtration of free carnitine and its reabsorption by renal tubules. For example, with progressive kidney disease, glomerular function decreases and plasma carnitine concentrations increase. Subsequent decline in renal function may lead to accumulation of acylcarnitines in plasma [38]. The methodology of our study did not involve simultaneous determination of the analysed metabolites in blood. We found a significant decrease in the level of acetylcarnitine excretion with urine during the first 3 days after OPs poisoning. Further studies are needed to answer the question whether this fact means inhibition of endogenous carnitine synthesis in the kidneys or generally reflects a systemic decrease in metabolism due to poisoning.

It is also an open question whether the decrease in the excretion of this metabolite is a consequence of the active involvement of carnitine in metabolic detoxification processes in response to OPs poisoning. This may be due to the fact that some researchers consider L-carnitine as an antioxidant [39]. This function of carnitine seems contradictory, due to the fact that carnitine enhances fatty acid metabolism, thereby promoting the formation of ROS in the electron transport chains of mitochondria [40]. However, there are publications indicating that L-carnitine determines NO formation and also activates the antioxidant enzymes superoxide dismutase (SOD) and catalase in response to neurotoxicity induced by 3-nitropropropionic acid [41]. It has been found that L -carnitine can directly chelate transition metals Fe^2+^ and Cu^+^, preventing their involvement in ROS formation [42]. L -carnitine is also able to reduce the formation of free radicals by inhibiting their enzymes xanthine oxidase and NADPH-oxidase, responsible for the formation of free radicals, which are of high biological relevance under various stress conditions [40]. In addition, carnitine is involved in maintaining mitochondrial integrity, including the mitochondrial electron transport chain, under stress conditions. In this regard, some researchers consider carnitine as a mitochondria-specific antioxidant responsible for maintaining mitochondrial integrity and regulating ROS and ROS signalling [40]. The protective effect of L -carnitine and its derivatives on the antioxidant systems of the body has been shown in various models of oxidative stress/toxicity induced by various toxicants and neurotoxic agents [42]. In view of the above facts, elucidation of the molecular mechanisms of carnitine and its derivatives under the influence of OPs is a promising task for future studies.

### Inosine

Inosine is present in all living organisms, including bacteria, plants in mammalian animals and humans. Inosine can be produced by gut bacteria. Inosine has been shown to activate peroxisome proliferator-activated receptor (PPAR)-gamma in human colon epithelial cells. Inosine can also be biosynthesised from inosinic acid by interaction with cytosolic purine-5'-nucleotidase. Inosine can be converted to hypoxanthine and ribose-1-phosphate by interaction with purine nucleoside phosphorylase [43]. Animal studies have shown that inosine has neuroprotective properties [44, 45], and exogenous inosine therapy enhances mucosal barrier functions to the development of experimental colitis in rodents [46].

The metabolism and excretion levels of inosine in rats were experimentally studied as early as 1982 [47]. After intravenous administration of twice labelled inosine to rats, the authors monitored the uptake and subsequent metabolism of purine and ribose molecules. More than 95% of inosine was eliminated from blood plasma within 5 min and 99% within 20 min. Approximately 50% of the total amount of 160 µmol was rapidly excreted into the liver and kidneys. The largest amount, 21 µmol/g wet weight, was excreted by the kidneys, about 10 times more than by the heart, lungs and liver. The lungs and heart accounted for only 3% [47]. Thus, it can be seen that excretion of inosine by the kidneys in rats is the most significant compared to other pathways.

In our study, the concentration of inosine in rat urine was significantly increased 1–3 days after exposure to OPs. At the same time, it is interesting to note that the concentration of hypoxanthine in the urine of animals during the same periods of observation, on the contrary, decreased. It is likely that this is a consequence of inhibition of the enzymatic activity of PNP, which metabolises inosine to hypoxanthine and guanosine to guanine, in each case forming ribose phosphate. This mechanism is known to be one of the enzymes of the nucleotide salvage pathway. It is also known that the de novo purine synthesis pathway can be interrupted by the action of some chemotherapeutic drugs, such as methotrexate [48]. In this regard, the question of how POX poisoning in any single poisoning model affects the mechanisms of purine biosynthesis remains open and requires further investigation.

### Hypoxanthine

Urinary excretion of hypoxanthine is a sensitive method for studying nucleotide metabolism, probably because it has a high renal clearance approaching the glomerular filtration rate [49] and also because hypoxanthine is relatively stable in the urine. Hypoxanthine excretion correlates well with creatinine excretion, whereas xanthine excretion does not [50]. In this regard, some researchers believe that the hypoxanthine/creatinine concentration ratio is a reasonable estimate of hypoxanthine excretion and correlates well with total excretion [51]. It is interesting to note that in our study, hypoxanthine concentration in rat urine did not correlate with creatinine excretion level and was significantly reduced within 1–3 days after exposure to OPs in all 3 poisoning models. We cannot explain this fact on the basis of the methodology used; further studies in this direction are needed.

The most frequent references to the increase in renal excretion of hypoxanthine as a result of the development of physiological dysfunction are found in the results of published studies. For example, experimental intermittent hypoxaemia (IH) in piglets results in increased urinary excretion of hypoxanthine (*p* < 0.04) after 60 min [52]. Interestingly, hypoxanthine has been shown in vitro to alter antioxidant defences and induce lipid peroxidation in rat kidney; however, in the presence of allopurinol and antioxidants, some of these changes in oxidative stress are prevented [53]. Hypoxanthine at a concentration of 10.0 μM increased catalase (CAT) and superoxide dismutase (SOD)activities in the renal cortex of 15-, 30- and 60-day-old rats, SOD activity in the renal medulla of 60-day-old rats and thiobarbituric acid (TBA-RS) levels in the renal medulla of 30-day-old rats compared to controls [53].

### 3-Hydroxy-methyl-butyrate (HMB)

HMB (beta-hydroxy beta-methyl butyric acid, 3-hydroxy-3-methylbutanoic acid) is a naturally occurring organic compound produced in many mammals and humans. The amino acid leucine, which is not incorporated into muscle protein, is eventually oxidised via intermediates such as HMB, which is actively involved in the regulation of skeletal muscle protein turnover [54–56].

Thus, HMB is synthesised in humans through the metabolism of L-leucine, a branched-chain amino acid. In healthy individuals, approximately 60% of dietary L -leucine is metabolised after a few hours, with an average of 5% (2–10%) of dietary L-leucine being converted to HMB [57, 58]. HMB is excreted by the kidneys, with approximately 10–40% of the ingested dose excreted unchanged in the urine The remaining 60–90% of the dose is retained in tissues or excreted as HMB metabolites [59].

Comparatively recently, HMB was thought to be detectable in plasma after oral administration of leucine in sheep and pigs, but not in Sprague–Dawley rats, which is the standard preclinical model [56]. Studies on the absorption, distribution, metabolism and excretion of radiolabelled leucine at a low dose (3 mg/kg) or high dose (1000 mg/kg) of 14C -leucine in rats have been published. The results indicate the appearance of 14C -HMB in plasma and urine of rats after oral administration of 14C-leucine. 14C-leucine appears in plasma as 14C-α-ketoisocaproic acid (KIC) with a slower time interval than 14C-HMB, the putative product of KIC [56]. Thus, the authors clearly demonstrated endogenous production of HMB from leucine in adult rats as in a standard preclinical animal model.

The results of our study showed a significant decrease in HMB concentration in the urine of rats at all observation periods after poisoning (1, 3 and 7 days) regardless of the poisoning model used. To explain this fact, it is necessary to carry out additional studies, in particular, to determine the mechanisms of inhibition of metabolic pathways of HMB biosynthesis.

## Conclusions

A novel methodology for the multi-target quantification of 11 metabolites in rat urine has been developed. The proposed assay procedure is a simple and reliable tool for urine metabolomic studies. Its applicability showed good performance in detecting changes in the content of metabolites in the urine of rats during their acute intoxication with paraoxon-ethyl in three models of acute exposure to OP. The results obtained in the present study indicate that all three models of acute intoxication of rats with paraoxon lead to significant changes in the content of ten metabolites in the urine of rats early after poisoning. Six of the ten metabolites may be recommended for further study as potential biomarkers of acute OP intoxication. The technique of organophosphorus compounds determination in urine can also be used to estimate the content of similar metabolites in human urine in acute poisoning by organophosphorus pesticides.

### Supplementary Information

Supplementary Material 1.

Supplementary Material 2.

Supplementary Material 3.

Supplementary Material 4.

## Data Availability

All datasets are presented in the main paper.

## Abbreviations

ALCAR, ALC: Acetylcarnitine
ATP: Adenosine triphosphate
CBDP: 2-(O-cresyl)-4H-1,3,2-benzodioxaphosphorin-2-oxide
Dilute-n-Shoot: A technique consisting of dilution of the original sample and subsequent analysis
ESI: Electro-spray ionisation
HMB: 3-Hydroxy-methylbutyrate
HPLC–MS/MS: High-performance liquid chromatography with tandem mass spectrometric detection method
MRM: Signal recording in multiple reaction monitoring mode
NO: Nitric oxide
IH: Intermittent hypoxaemia
IMP: Inosine monophosphate
IS: Internal Standard
OP (s): Organophosphate (s)
POX: Paraoxon-ethyl
ROS: Reactive oxygen species
SD: Standard deviation
SOD: Superoxide dismutase
Thr: Treonine
