# Supplementary material for: Quantification of 11 metabolites in rat urine after exposure to organophosphates
Source: Lab Anim Res. 2024 Jun 6;40:23. doi: 10.1186/s42826-024-00209-3 (PMC11155157; doi:10.1186/s42826-024-00209-3)
Supplement: Supplementary file 1 — Supplementary Material 1. [file 42826_2024_209_MOESM1_ESM.docx]

**Additional file 1** **(Figure 1)**

Mass chromatograms of metabolites in rat urine: a-3-methylhistidine; b- L-threonine; c-creatine; d-creatinine; e-lactic acid; f- acetylcarnitine.

**a**

**b**

**c**

**d**

**e**

**f**
