# Supplementary material for: Quantification of 11 metabolites in rat urine after exposure to organophosphates
Source: Lab Anim Res. 2024 Jun 6;40:23. doi: 10.1186/s42826-024-00209-3 (PMC11155157; doi:10.1186/s42826-024-00209-3)
Supplement: Supplementary file 2 — Supplementary Material 2. [file 42826_2024_209_MOESM2_ESM.docx]

**Additional file 2** **(Figure 2)**

Mass chromatograms of metabolites in rat urine: g- inosine; h- hypoxanthine; i- adenine; j- 3-hydroxybutyrate; k - 2-hydroxybutyrate; l- deuterated (D-3) 2-(2-carboxyethyl)-1,1,1,1-trimethylhydrazinium.

**g**

**h**

**i**

**j**

**k**

**l**
