# Supplementary material for: Quantification of 11 metabolites in rat urine after exposure to organophosphates
Source: Lab Anim Res. 2024 Jun 6;40:23. doi: 10.1186/s42826-024-00209-3 (PMC11155157; doi:10.1186/s42826-024-00209-3)
Supplement: Supplementary file 3 — Supplementary Material 3. [file 42826_2024_209_MOESM3_ESM.docx]

**Additional file 3** **(Figure 3)**

Dynamics of metabolite content changes in rat urine: a - 3-methylhistidine; b - threonine; c - creatine; d - creatinine; e - lactic acid; f - acetyl-carnitine. The vertical bars at each point on the graph denote the interquartile range.

**a**


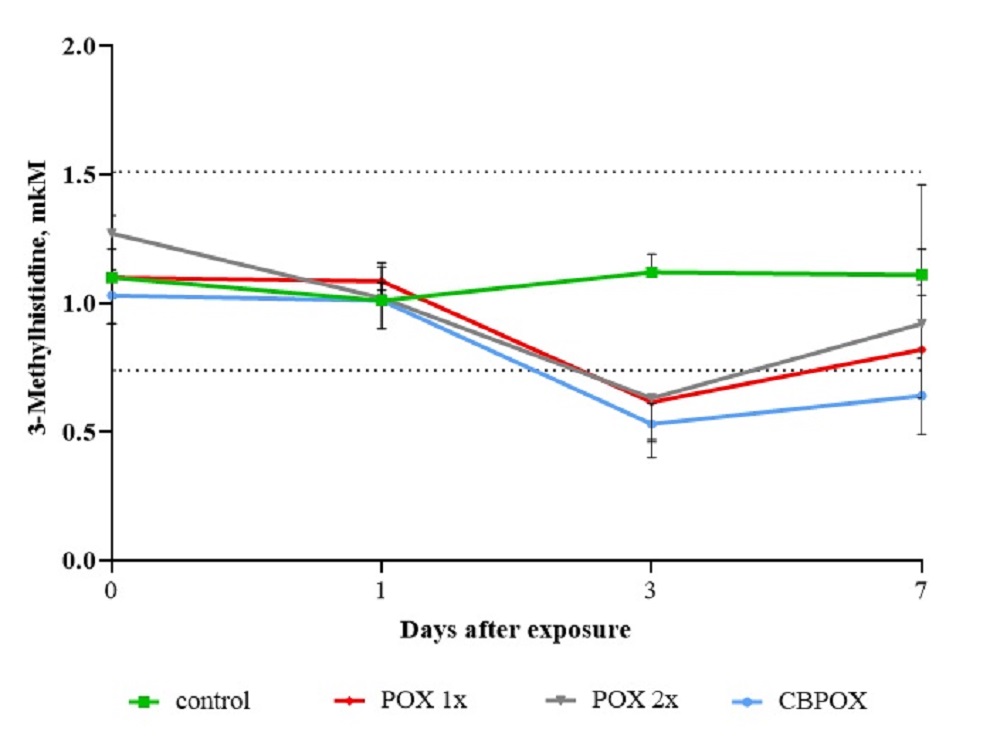


**b**


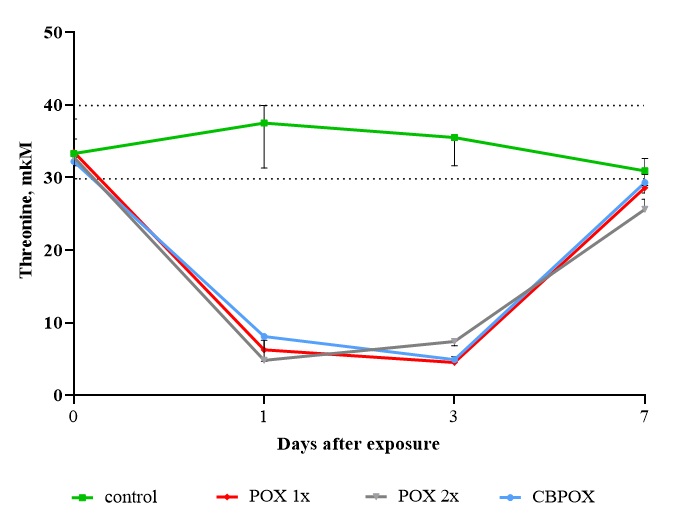


**c**


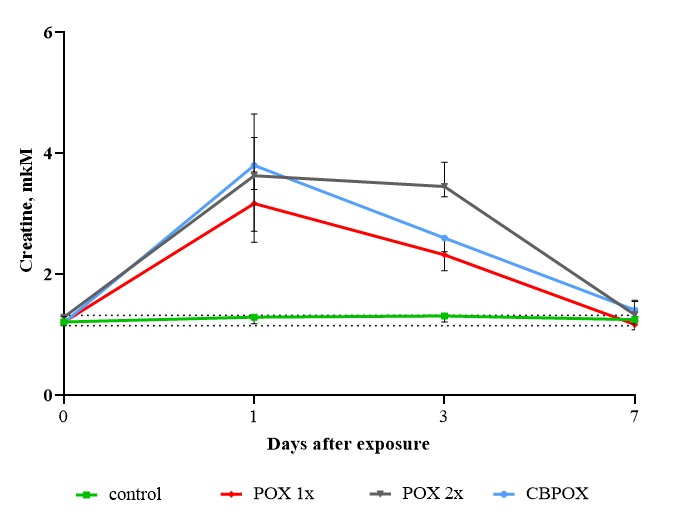


**d**


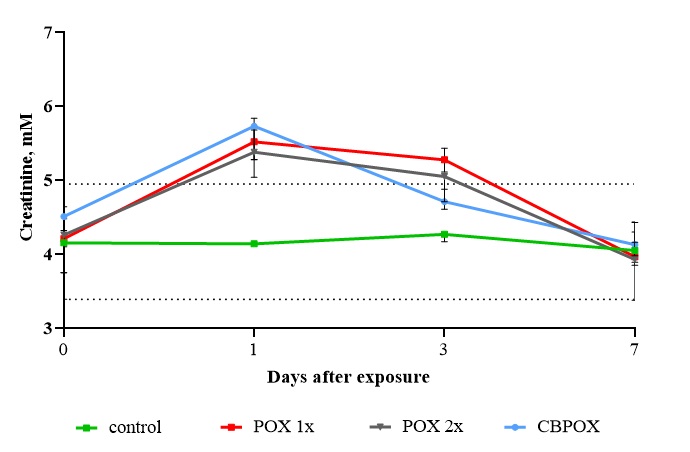


**e**

**
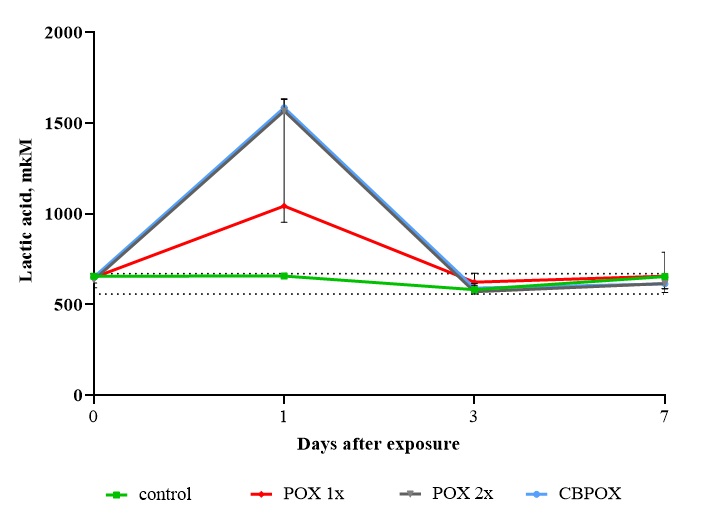
**

**f**

**
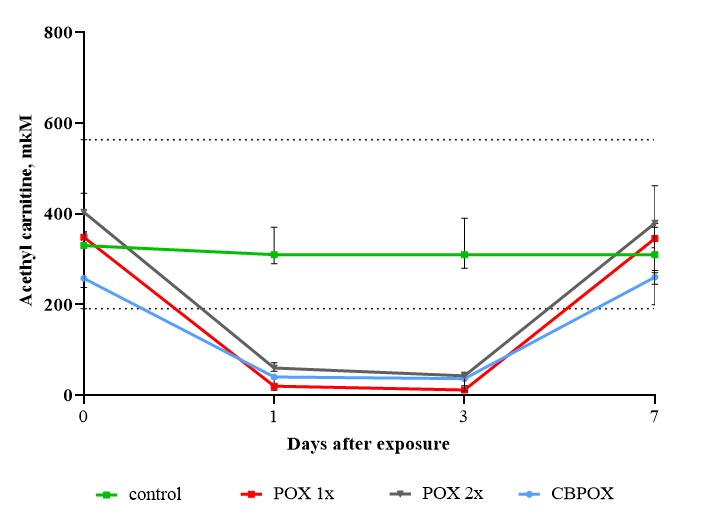
**
