# Supplementary material for: Quantification of 11 metabolites in rat urine after exposure to organophosphates
Source: Lab Anim Res. 2024 Jun 6;40:23. doi: 10.1186/s42826-024-00209-3 (PMC11155157; doi:10.1186/s42826-024-00209-3)

**Additional file 4** **(Figure 4)**

Dynamics of changes in the content of metabolites in rat urine: g - inosine; h - hypoxanthine; i - adenine; j - 3-hydroxymethylbutyrate (3-HMB); k - 2-hydroxymethylbutyrate (2-HMB). The vertical bars at each point on the graph denote the interquartile range.

**g**


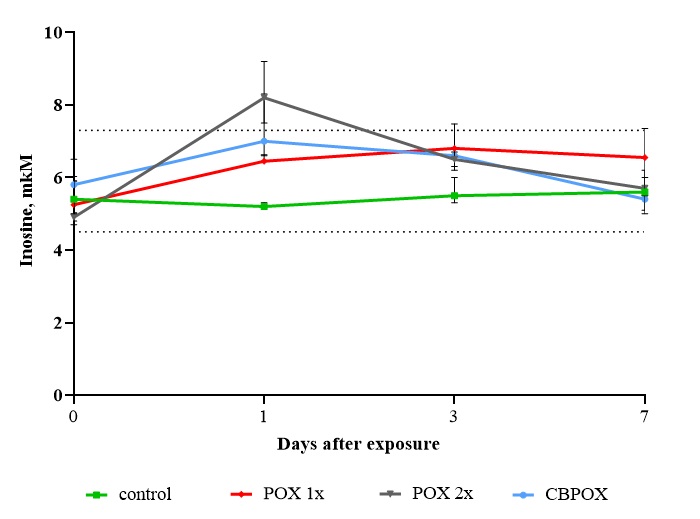


**h**


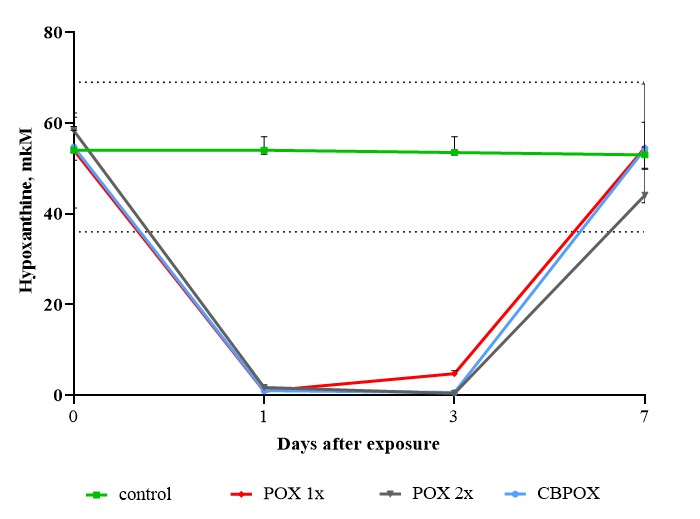


**i**


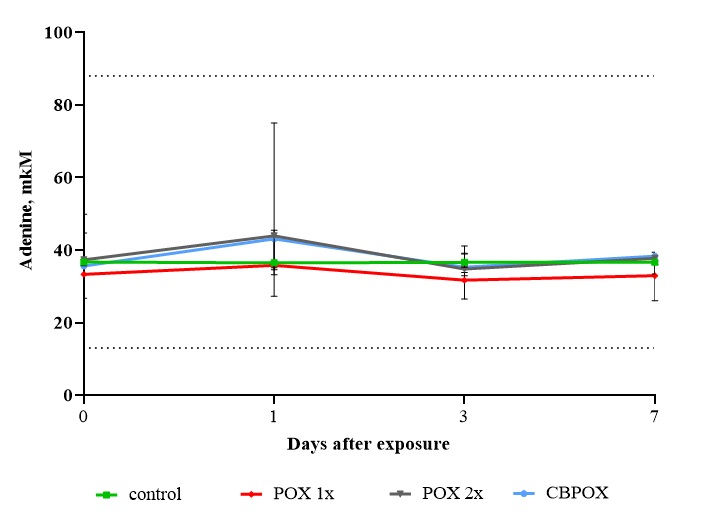


**j**


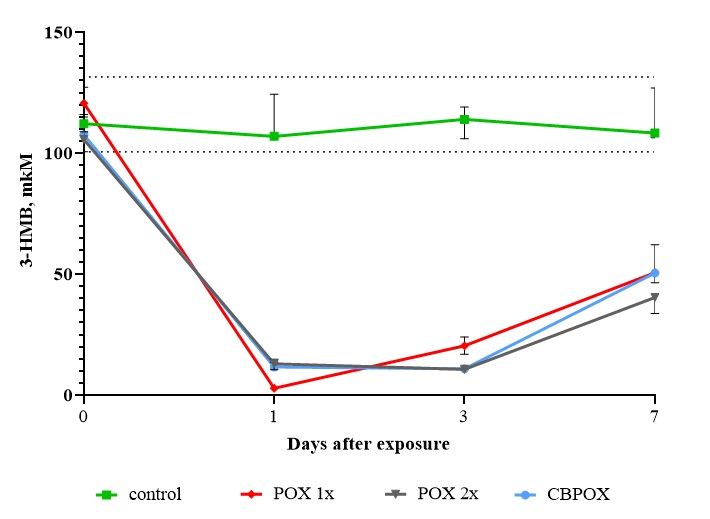


**k**


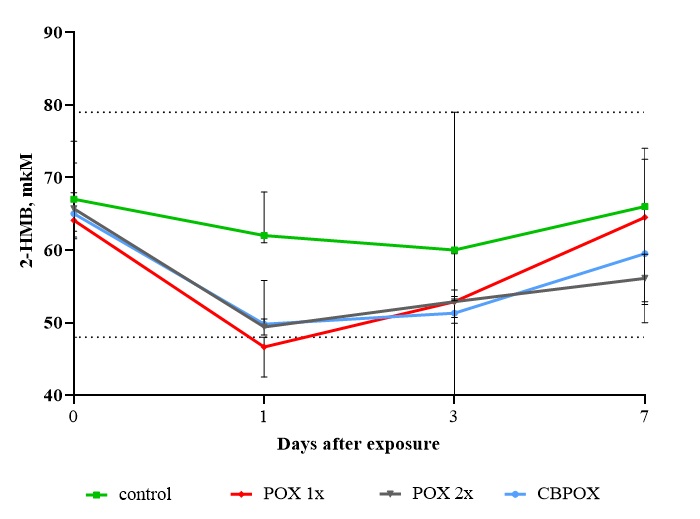

Supplement: Supplementary file 4 — Supplementary Material 4. [file 42826_2024_209_MOESM4_ESM.docx]
